# Modulation of CREB3L2-ATF4 heterodimerization via proteasome inhibition and HRI activation in Alzheimer’s disease pathology

**DOI:** 10.1038/s41419-025-07586-0

**Published:** 2025-03-31

**Authors:** Krystal Herline-Killian, Michaela M. Pauers, Jessica E. Lipponen, Michael A. Zrzavy, Cláudio Gouveia Roque, Ethan P. McCurdy, Kyung Min Chung, Ulrich Hengst

**Affiliations:** 1 The Taub Institute for Research on Alzheimer’s Disease and the Aging Brain, Vagelos College of Physicians and Surgeons, Columbia University, New York, NY USA; 2 Doctoral Program in Neurobiology and Behavior, Columbia University, New York, NY USA; 3 Graduate Program in Pathobiology and Mechanisms of Disease, Vagelos College of Physicians and Surgeons, Columbia University, New York, NY USA; 4 Integrated Program in Cellular, Molecular and Biomedical Studies, Vagelos College of Physicians and Surgeons, Columbia University, New York, NY USA; 5 Department of Pathology & Cell Biology, Vagelos College of Physicians and Surgeons, Columbia University, New York, NY USA

**Keywords:** Cell death in the nervous system, Stress and resilience, Cellular neuroscience

## Abstract

Alzheimer’s disease (AD) pathology includes transcriptional changes in the neurons, which are in part caused by the heterodimerization of two stress response transcription factors, CREB3L2 and ATF4. We investigated the role of proteasome inhibition and the eIF2α-kinase HRI in the formation of CREB3L2-ATF4 in neurons exposed to soluble oligomeric Aβ_42_. While HRI activation increased ATF4 expression, it decreased CREB3L2 and CREB3L2-ATF4 levels. Proteasome inhibition, induced by Aβ_42_, leads to increased levels of both transcription factors in the nucleus. These findings suggest that CREB3L2 levels are normally kept low due to rapid degradation, but proteasome inhibition in response to Aβ_42_ disrupts this balance, increasing CREB3L2 and heterodimer levels. Activation of HRI not only reduced CREB3L2 and heterodimer levels but also prevented the transcriptional dysregulation of a CREB3L2-ATF4 target, SNX3. Our results suggest that manipulating the HRI pathway during proteasome inhibition could help restore normal gene expression in the context of AD-related protein accumulation.

## Introduction

Alzheimer’s disease (AD) is a debilitating neurodegenerative disease characterized among others, by the progressive accumulation of β-amyloid 1-42 (Aβ_42_) and tau protein aggregates, widespread transcriptional changes throughout the brain, and ultimately, profound cognitive deficits [1–3]. Identifying mechanisms driving these transcriptional changes might provide novel targets to therapeutically normalize gene expression in AD [4]. We discovered that a unique heterodimer of two bZIP transcription factors, cyclic AMP-responsive element-binding protein 3-like protein 2 (CREB3L2) and activating transcription factor 4 (ATF4), is present in AD brain [5–7]. This heterodimer is linked to up to 50% of gene expression changes in AD brain, and its induction in neurons is sufficient to elicit AD-typical neuronal changes, including the hyperphosphorylation and secretion of tau, increased Aβ_42_ over Aβ_40_ production, and disrupted expression of components of the retromer [6], which functions in endosomal-lysosomal trafficking and is defective in AD brain [8, 9]. Most importantly, the prevention of dimer formation rescues neurons from Aβ_42_-induced cell death [6]. CREB3L2-ATF4 is rarely seen in health control brains [6], and the mechanisms governing its formation and or stability specifically in Aβ_42_-exposed neurons or the AD brain remain unknown.

The levels of stress response transcription factors, such as ATF4, are under tight translational control [10]. *Atf4* and *Creb3l2* belong to a class of mRNAs, whose translation is positively controlled as part of the integrated stress response (ISR) [11]. The ISR is a network of cellular signaling pathways that get activated in response to a variety of stressors to reestablish cellular homeostasis [12]. These pathways converge in the phosphorylation of the α-subunit of the eukaryotic initiation factor 2 (eIF2α) and the resulting synthesis of stress response transcription factors. Currently, it is unclear which of the eIF2α kinases (PERK, PKR, GCN2, HRI) are activated in response to Aβ_42_ in neurons and required for the synthesis and subsequent dimerization of ATF4 and CREB3L2. There is a paucity of data on the role of the ISR kinase HRI in the brain because it was once thought to only function in balancing globin synthesis with heme levels in red blood cells during hemoglobin synthesis [13]. More recently, HRI has received renewed interest as the ISR kinase responsible for phosphorylating eIF2α during proteasome inhibition [14–17]. Furthermore, HRI has now been identified in the brain and functions in spine growth, memory consolidation, and postsynaptic activity [18, 19]. In addition to translation control, post-translational mechanisms, especially ubiquitination and proteasomal degradation, control the abundance of ATF4 and CREB3L2. ATF4 contains a β-TrCP degron, which unless multiphosphorylated causes its rapid degradation [20]. Similarly, CREB3L2 is rapidly ubiquitinated and degraded by the proteasome under non-stress conditions [21]. How these two regulatory mechanisms, translational activation downstream of eIF2α and ubiquitin-dependent proteasomal degradation, affect the formation and abundance of the CREB3L2-ATF4 heterodimer in Aβ_42_-exposed neurons remains unexplored.

Here, we report that activation of the ISR is not sufficient for the formation of the CREB3L2-ATF4 but that concurrent inhibition of the cytoplasmic proteasome is required as well. Activation of HRI, which can be activated in response to inhibition of the proteasome, strongly induces ATF4 levels, but its prolonged activation reduced CREB3L2 expression. Consequently, we find that sustained HRI activation prevents heterodimer formation and signaling.

## Materials and Methods

### Primary culture of rat embryonic neurons

Timed-pregnant Sprague-Dawley rats were housed in the barrier facility at the Columbia University Institute of Comparative Medicine. All animal procedures were approved by the Institutional Animal Care and Use Committee at Columbia University. Primary cortical neurons were harvested from E15-E18 rat embryos and cell dissociation was performed by incubating with TrypLE Express Enzyme (Thermo Fisher Scientific) for 15 minutes in a water bath at 37°C followed by repeated pipetting through a fine pipette tip. Neurons were plated on substrates coated with 0.1 mg ml^-1^ poly-D-lysine (MilliporeSigma) and 2 mg ml^-1^ laminin (Bio-Techne) and then grown in Neurobasal medium supplemented with 10% fetal bovine serum (heat inactivated, certified One Shot FBS; Thermo Fisher Scientific), 2 mM L-glutamine (Gibco), 1 mM sodium pyruvate (Gibco), and 50 U ml^-1^ penicillin-streptomycin (Gibco) and placed in a humified atmosphere of 5% CO_2_ at 37°C. The next day, the medium was changed to Neurobasal containing 1x B27 (Gibco) and 2 mM L-glutamine. Half-medium changes were performed every 3-4 days and maintained in a 37°C, 5% CO_2_ humidified atmosphere until DIV7-8.

### Cell culture

HEK293T (ATTC) were cultured in DMEM supplemented with 10% FBS and 2 mM L-glutamine in a humidified atmosphere of 5% CO_2_ at 37 °C. Cells were passaged at 80–95% confluency. Experiments were performed when cells were at a 75-85% confluency.

### Lentivirus constructs, preparation, and transduction

Lentivirus expression plasmids shControl and shHRI (shHRI sequence: 5’-TGCTGTTGACAGTGAGCGCCAAGACAGAGTTCCCATTCAATAGTGAAGCCACAGATGTATTGAATGGGAACTCTGTCTTGTTGCCTACTGCCTCGGA-3’) were obtained from transOMIC technologies. The original mCMV promoter was replaced with the human ubiquitin C (hUbC) promoter for expression in primary neurons using the In-Fusion HD Cloning Plus kit (Takara Bio). Lentiviruses were produced by transfecting HEK293T cells plated in a 100-mm plate at 85–90% confluency with the lentiviral plasmid and packaging plasmids (pCMVΔ R8.9 and pHCMV VSVg) using Lipofectamine 3000 transfection reagent (Thermo Fisher Scientific). Cells were changed into neuronal growth medium after 12–16 h. Virus containing medium was passed through a 0.45 μm PES filter 33–36 h later, aliquoted, and stored at −80°C. Titers for every batch were determined using a qPCR lentivirus titration kit (Applied Biological Materials). Primary cortical neurons were infected on DIV1 with shHRI or shControl at an MOI 20. Experiments were performed on DIV8 unless indicated otherwise.

### Generation of GFP-tagged CREB3L2

cDNA containing *H. sapiens* CREB3L2 open reading frame was acquired from Genecopoeia (NCBI Reference sequence: NM_194071; product #EX-H2495-M01) and subcloned into pEGFP-C1 (Clontech). EcoRI and BamHI restriction sites were introduced by PCR with CloneAmp HiFi polymerase (Takara), and end products were validated by Sanger sequencing. In-fusion cloning was used to mutate the S1P cleavage site (In-Fusion HD EcoDry Cloning Kit, Takara).

### Aβ_42_ peptide oligomerization

Lyophilized synthetic Aβ_42_ peptides (Bachem, H-1368) were dissolved to 1 mM in ice-cold 100% 1,1,1,3,3,3-hexafluoro-2-propanol (MilliporeSigma) by multiple rounds of pipetting, aliquoted, and spun in SpeedVac (Savant Instruments) for 30 minutes. The peptide films were resuspended in dimethyl sulfoxide (DMSO; MilliporeSigma) and further diluted with Ham’s F12 medium (Thermo Fisher Scientific), and incubated overnight at 4 °C, as described previously [22]. Aβ was added to dissociated cultures at a concentration of 250 to 750 nM to accommodate batch-to-batch variations. Vehicle controls consisted of a DMSO/F12 mixture, which was similarly incubated overnight.

### Drug treatments

HEK293T and cortical neurons were treated with 1.27 µM thapsigargin (MilliporeSigma), 12 µM BtdCPU (EMDMillipore), 15 µM nelfinavir (MilliporeSigma), 6.8 µM bortezomib (MilliporeSigma), or DMSO as a control for 5 h, unless otherwise stated.

### Nuclear fractionation

For nuclear fractionation, cells were plated in 100 mm or 60 mm tissue cultured plates. For collecting a nuclear enriched fraction from Aβ_42_- or drug-treated samples, cells were washed in HBSS and collected in ice-cold HBSS supplemented with Pierce protease inhibitors (Thermo Fisher Scientific). Samples were collected into pre-chilled microcentrifuges tubes by scraping. Cells were spun at 3,000 RPM for 5 minutes at 4°C. Supernatant was discarded, and the pellets were gently suspended in 200 µl hypotonic buffer (20 mM Tris-HCl, pH 6.8, 10 mM NaCl, 5 mM EDTA) supplemented with HALT protease and phosphatase inhibitor cocktail (Thermo Fisher Scientific), by gently pipetting up and down several times. Samples were incubated on ice for 15 minutes and then transferred to a new pre-chilled microcentrifuge tube. 15 µl of a 10% Triton-X solution was added to the cells, immediately vortexed for 10 seconds, and then spun down for 10 minutes at 10,000 RPM at 4 °C. The supernatant was collected as the cytoplasmic enriched fraction. The pellet-containing nuclei were suspended in 30 µl RIPA buffer (Thermo Fisher Scientific) supplemented with HALT phosphatase and protease inhibitor cocktail (Thermo Fisher Scientific) and incubated for 30 minutes on ice with vortexing every 10 minutes. Samples were centrifuged at 14,000 RPM for 30 minutes at 4 °C and the supernatant containing the nuclear fraction was collected and protein concentration was measured by BCA protein assay.

### Coimmunoprecipitation in primary neuronal culture

Protein A magnetic beads (New England Biolabs) were washed three times in RIPA buffer (Thermo Fisher Scientific). 1 µg of monoclonal antibody ATF4 (D4B8, Cell Signaling Technology) and IgG (DA1E, mAb IgG XP® Isotype Control) diluted in RIPA buffer was added to 40 µl of beads for each condition, mixed for 2 h at 4 °C, and washed three times with RIPA. Antibodies were covalently cross-linked to the protein A beads using bis(sulfosuccinimidyl)suberate (BS3; Thermo Fisher Scientific). During this step, neurons were freshly lysed with RIPA buffer and the resulting protein was quantified by BCA assay. Equal amounts of antibody-bead conjugates were mixed with 500 µg lysates overnight at 4 °C with constant rotation. Beads were washed three times at 4 °C with RIPA buffer. Immunoprecipitates were eluted in 50 μl of 0.2 M glycine buffer (pH 2.5) and allowed to react for 5 minutes at 4 °C with rotation after a short vortexing step. Eluates were transferred to a new tube, and the elution protocol was repeated. Pooled eluates were neutralized by the addition of 20 μl of 1 M Tris-Cl (pH 9.0). An aliquot of eluate was then mixed with 4x Laemmli buffer to be analyzed by western blot and the remainder was immediately stored at −20 °C. CREB3L2 levels were quantified by immunoblot using anti-CREB3L2 antibodies (HPA015068, Atlas Antibodies).

### Immunoblotting

Cells were washed with ice-cold HBSS, scraped, and collected in ice-cold RIPA buffer supplemented with HALT protease and phosphatase inhibitor cocktail. Extracts underwent end-over-end rotation at 4 °C for 30 minutes, followed by centrifugation at 14,000 RPM for 30 minutes at 4 °C. Supernatants were collected, and protein concentration was measured using BCA assay (Pierce). Lysates were mixed in Laemmli sample buffer (Biorad) containing 100 mM dithiothreitol (DTT) and heated to 95 °C for 5 minutes. Samples were resolved by SDS-PAGE using NuPage MOPS SDS buffer and NuPAGE electrophoresis system (Thermo Fisher Scientific), followed by wet transfer using the XCell II Blot Module (Thermo Fisher Scientific) to 0.2 μm pore-sized nitrocellulose membranes (GE Healthcare). Membranes were blocked for 1 h with 5% milk in TBS containing 0.1% Tween (TBST) and washed in TBST. Nitrocellulose membranes blotted overnight at 4°C using the following primary antibodies: anti-CREB3L2 (1:1000; HPA015068, Atlas Antibodies), CREB3L2 (1:1000; MABE1018, MilliporeSigma), anti-ATF4 (1:1000; #11815, Cell Signaling Technology), anti-SNX3 (1:1000; ab56078, Abcam), anti-S2P (1:1000; AB140594, Abcam), anti–β-actin (1:10,000; #3700, Cell Signaling Technology), and anti-HDAC1 (1:10,000; ab109411, Abcam). The next day, membranes were washed with TBST, and incubated with IgG anti-mouse or anti-rabbit, HRP secondary antibodies (Thermo Fisher Scientific) for 1 h at room temperature. Membranes were washed, and signals were visualized on KwikQuant Imager (Kindle Biosciences) using 1-Shot Digital-ECL Substrate Solution (Kindle Biosciences) and quantified using Fiji/ImageJ. Replicates for an experiment were always run in a random order on each blot to minimize position effects. Full length, uncropped original western blot s are available as a Supplementary File.

### Proximity ligation assays and subsequent quantification

Cells grown on glass-bottom dishes (MatTek Corporation) were washed 3x with PBS fixed in 4% paraformaldehyde and 4% sucrose in PBS (pH 7.4) for 15 minutes at room temperature. After PBS washes, neurons were permeabilized with 0.2% Triton X-100 in PBS for 10 minutes. Blocking was performed for 1 h with 5% heat-inactivated goat serum (Gibco) diluted in PBS, and primary antibodies (rabbit anti-CREB3L2, 1:300, HPA015534 (Atlas antibodies); mouse anti-ATF4, 1:300, 2B3 WH0000468M1 (MilliporeSigma)), were prepared in blocking solution and then incubated overnight at 4°C. PLA protocol was performed using Duolink In situ Red Detection reagents (MilliporeSigma) with TBS and TBST. All incubations were performed in a humidity chamber and in a hybridization oven (Hoefer Red Roller II). After the last wash step of the Duolink protocol, neurons were counterstained with Alexa Fluor 488–conjugated βIII-tubulin antibody (1:500; BioLegend) diluted in a 1% TBS for 1 h and preserved in Duolink In Situ Mounting Medium with DAPI (MilliporeSigma). Samples were imaged using an Axio Observer Z1 microscope (Zeiss) on 63x oil objective and Zen Blue 2.1 software (Zeiss) or, when visualizing nuclear events, using an Andor BC43 benchtop confocal microscope on 60x oil objective and Fusion software with subsequent image processing in ImarisViewer. Imaging settings were kept constant between conditions. PLA puncta for CREB3L2-ATF4 were counted manually for nuclear events or, for total somatic events, quantified using the published speckle counting pipeline from the cell imaging analysis software CellProfiler [23]. When quantifying total somatic events, puncta were normalized to the area of soma, which had been manually traced using the IdentifyObjectsManually module in a blinded fashion.

### Proteasome assay

Previously collected Aβ_42_- or vehicle-treated neurons stored at −80 °C were thawed on ice. Cells were lysed in a lysis buffer (50 mM HEPES, 10 mM NaCl, 1.5 mM MgCl_2_, 1 mM EDTA, 0.5% Triton X) supplemented with 2 mM ATP, and resulting lysates were collected and stored on ice. Equal volumes of lysate were added to assay buffer (50 mM HEPES, 10 mM NaCl, 1.5 mM MgCl_2_, 1 mM EDTA, 2 mM ATP, 5 mM DTT, and 10 mM of a fluorogenic substrate [Suc-LLVY-AMC (Enzo Life Sciences), Z-ARR-AMC (EMD Millipore), or Z-LLE-AMC (EMD Millipore)) in 96-well black/clear bottom plates (Thermo Fisher Scientific). For the assay buffer, ATP, DTT, and the fluorogenic substrate were added fresh. Conditions were performed in duplicates. All samples were assayed with and without proteasome inhibitor (bortezomib or epoxomycin (Enzo Life Sciences)) to determine background AMC cleavage by other proteases in the sample. Plates were kept in the dark at 37 °C for 2 h then read at Ex/Em = 350/460 nm on a fluorescent plate reader (Tecan). Proteasome activity was determined for a single sample by subtracting the relative fluorescence of a bortezomib/epoxomicin treated sample from that of the same sample not treated with inhibitor.

### Statistical analysis

Statistical analysis was performed using GraphPad Prism 10.4.0. When comparing the means of two groups, a paired or unpaired *t*-test was performed depending on the experimental design (see figure legends). When comparing the means of three independent groups, a one-way analysis of variance (ANOVA) followed by Dunnett’s multiple comparisons test or Tukey’s multiple comparisons test was performed depending on the experimental design. When comparing the means of multiple groups encompassing two independent variables, two-way ANOVA with Holm-Šídák multiple comparison tests were performed. A number of biological replicates is indicated in each figure legend.

## Results

### ER stress alone is insufficient to induce CREB3L2-ATF4 heterodimerization in neurons

**Fig. 1:**
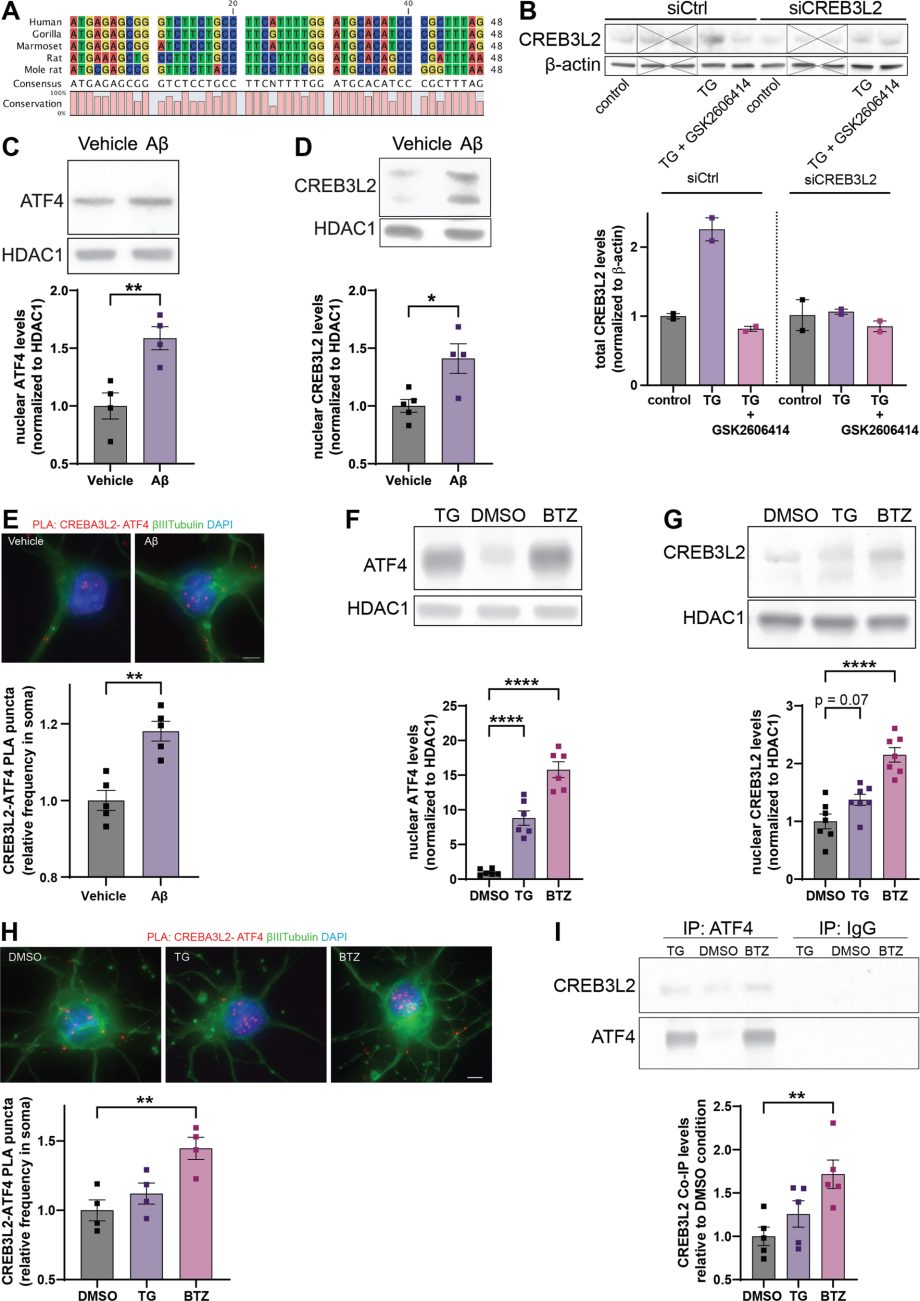
Proteasome inhibition induces formation of CREB3L2-ATF4 complexes in neurons. **A** The 5’ uORF of CREB3L2 is conserved across various higher order vertebrates. **B** Induction of ER stress causes PERK-dependent CREB3L2 synthesis. Cortical neurons were cultured for 10 DIV and treated with thapsigargin (1 μM) and/or GSK2606414 (5 μM) for 5 h. Scrambled control or CREB3L2-targeting siRNA was applied for 36 h before treatments. *n* = 2 replicates; unrelated lanes crossed out. C-D Immunoblot analysis of nuclear ATF4 (**C**) and CREB3L2 (**D**) levels in cortical neurons treated with vehicle or Aβ_42_ for 36 h. Means ± SEM of *n* = 4-5 independent biological replicates, normalized to HDAC1 expression; *p = 0.0153, **p = 0.0082, unpaired t-test. **E** PLA for CREB3L2-ATF4 in cortical neurons treated with Aβ_42_ for 8 h. Means of means ± SEM of *n* = 5 independent biological replicates, 48-50 optical fields per condition. Number of puncta normalized to area of neuronal soma. Unpaired, two-tailed t-test. **p < 0.01. Scale bar, 5 µm. F-G Immunoblot analyses of ATF4 (**F**) and CREB3L2 (**G**) levels in nuclear lysates of vehicle (DMSO), thapsigargin (TG), or bortezomib (BTZ) treated cortical neurons. Means ± SEM of *n* = 7 independent biological replicates, normalized to HDAC1. One-way ANOVA followed by Dunnett’s multiple comparison test. ****p < 0.0001. **H** PLA of CREB3L2-ATF4 in cortical neurons treated with vehicle (DMSO), bortezomib (BTZ), or thapsigargin (TG) for 5 h. Means of means ± SEM of *n* = 4 independent biological replicates, 40 optical fields per condition. Number of puncta normalized to area of neuronal soma. One-way ANOVA followed Dunnett’s multiple comparison test. *p < 0.05. Scale bar, 5 µm. **I** Co-immunoprecipitation of CREB3L2 with anti-ATF4 antibodies or control IgG from lysates of cortical neurons treated with vehicle (DMSO), thapsigargin (TG), or bortezomib (BTZ) for 5 h. Means ± SEM of *n* = 5 independent biological replicates. One-way ANOVA followed by Dunnett’s multiple comparison test. **p < 0.001.

The AD-linked peptide soluble oligomeric Aβ_42_ induces the formation of a pathogenic heterodimer between the stress response transcription factors CREB3L2 and ATF4 in neurons [6], but the mechanisms controlling their expression and heterodimerization remain unclear. Upon activation of the ISR, eIF2α-phosphorylation selectively increases the translation of mRNAs containing an upstream open reading frame (uORF) in their 5’-untranslated region (UTR), such as *Atf4* [24]. CREB3L2 belongs to the OASIS family of ER stress transducers, whose expression is translationally controlled by cellular stress [11, 25]. Despite being an OASIS family member, being translated in response to ER stress, and containing several conserved potential uORFs in its 5’UTR (Fig. 1A), *Creb3l2* mRNA has not yet been formally identified as an effector of the ISR. Induction of ER stress by treating cortical neurons with the sarcoplasmic and endoplasmic reticulum Ca^2+^-ATPase (SERCA) inhibitor thapsigargin resulted in a marked increase in CREB3L2 expression as determined by immunoblot, which was abolished by transfecting the neurons with an siRNA targeting *Creb3l2* or by treating the neurons with GSK2606414, a selective PERK inhibitor [26] (Fig. 1B). This formal confirmation that both *Atf4* and *Creb3l2* are ISR effector mRNAs, prompted us to identify the eIF2α kinase(s) involved.

We treated cortical neurons for 36 h with soluble oligomeric Aβ_42_ and determined the levels of ATF4 and CREB3L2 nuclear lysates by immunoblotting. Aβ_42_ induced a significant increase for both transcription factors in neuronal nuclei (Fig. 1C, D), as well as a marked increase in CREB3L2-ATF4 heterodimers as detected by proximity ligation assay (PLA) (Fig. 1E). To test whether ER stress was similarly sufficient to induce CREB3L2-ATF4 signaling, we treated primary cortical neurons with thapsigargin for 5 h. Nuclear ATF4 levels were induced to a much greater extent than with Aβ_42_-treatment, while the induction of ER stress failed to cause a significant increase in CREB3L2 levels (Fig. 1F, G). Because heterodimer formation is not necessarily driven by changes in the protein levels, we investigated if induction of ER stress would lead to an increase in CREB3L2-ATF4 heterodimers, even in the absence of CREB3L2 upregulation. With two complementary approaches, proximity ligation assay (PLA) and co-immunoprecipitation (co-IP), we did not detect any induction of CREB3L2-ATF4 dimerization in response to thapsigargin (Fig. 1H, I).

### Proteasome inhibition triggers CREB3L2-ATF4 heterodimerization

**Fig. 2:**
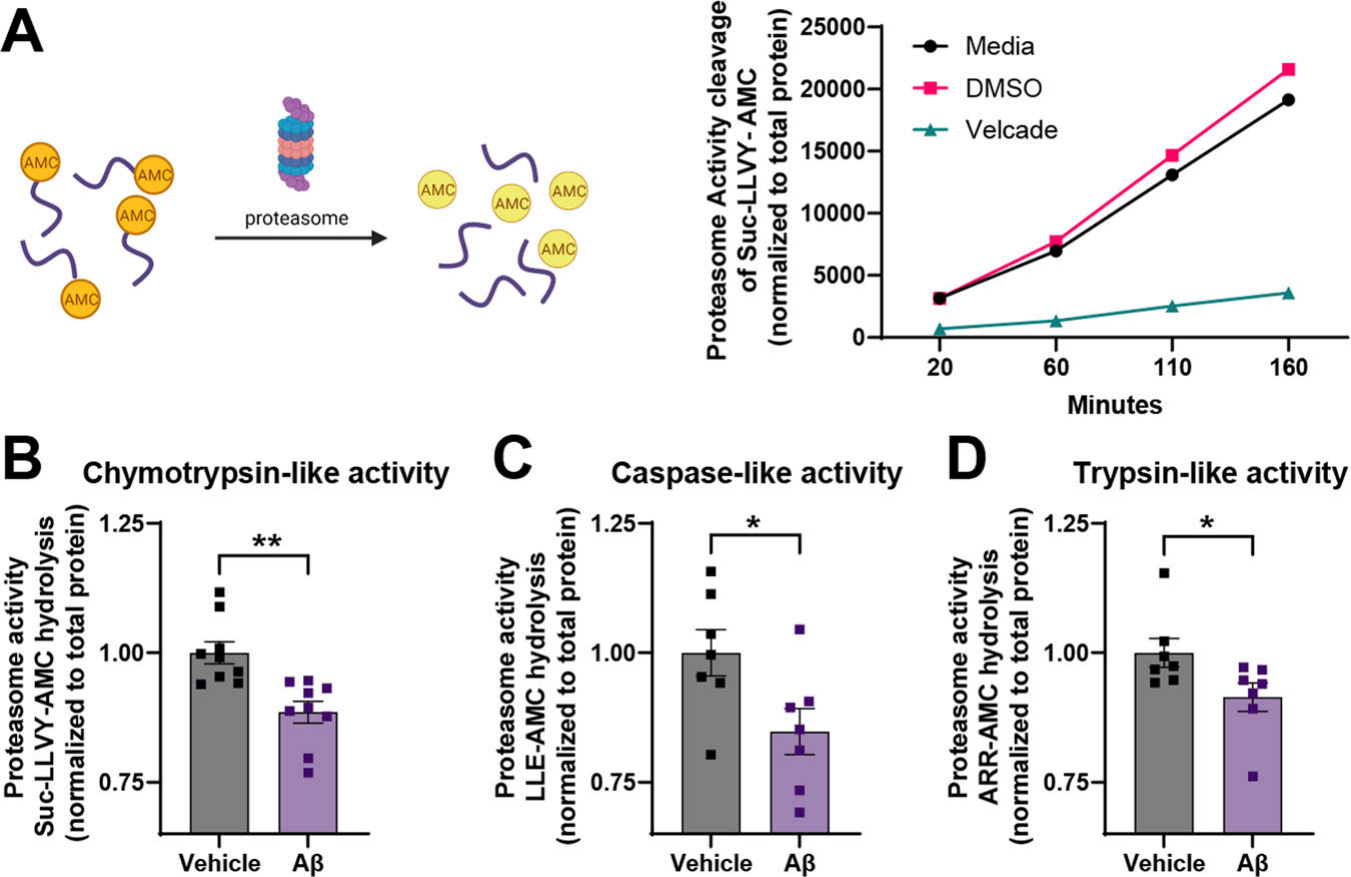
Soluble oligomeric Aβ_42_ causes reduced proteasome activity in neurons. **A** Validation of the proteasome activity assay. Proteasome activity assays on lysates of cortical neurons treated with vehicle or Aβ_42_ for 8 hours. Peptidase activity of the 20S proteasome was measured at 120 minutes using specific fluorogenic substrates (50 µM): Suc-LLVY-AMC (chymotrypsin-like; **B**), Z-LLE-AMC (caspase-like; **C**), or Z-ARR-AMC (trypsin-like; **D**). Means ± SEM of *n* = 7 (B) or 9 (**C**, D) independent biological replicates. Unpaired, two-tailed t-tests. *p < 0.05; **p < 0.01.

CREB3L2 and ATF4 are also known to be affected by the inhibition of the proteasome [11, 21, 27], and indeed, proteasome inhibition with bortezomib (BTZ) for 5 h led to an increase in both ATF4 and CREB3L2 protein levels (Fig. 1F, G). Levels of the heterodimer complex were also increased upon BTZ treatment as visualized by PLA and co-IP (Fig. 1H, I), indicating that an increase in CREB3L2-ATF4 heterodimer levels requires proteasome inhibition. In AD, proteasome activity is impaired [28–30], and Aβ_42_ inhibits the activity of the proteasome [29, 31–36]. To determine if extracellular soluble oligomeric Aβ_42_ in our experimental system inhibited the proteasome, we measured chymotrypsin-, trypsin, and caspase-like activity of the proteasome and confirmed that proteasome activity was significantly reduced in neurons treated with Aβ_42_ (Fig. 2).

### Aβ_42_ induces S2P-dependent cleavage of CREB3L2

**Fig. 3:**
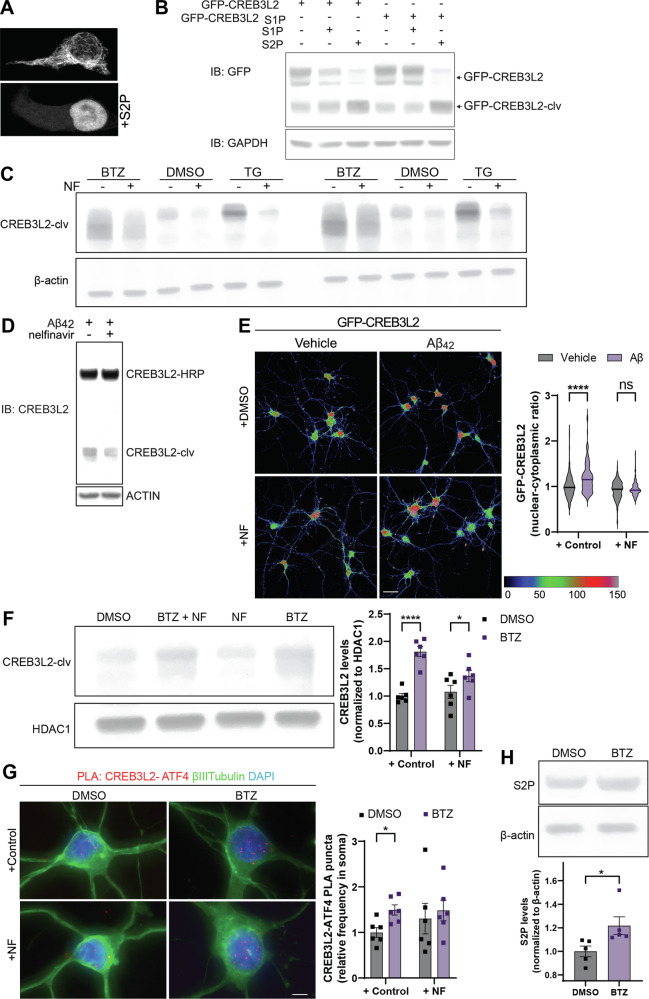
Proteasome inhibition activates S2P-dependent cleavage and nuclear localization of CREB3L2. **A** Subcellular distribution of a GFP-CREB3L2_S1P_ (full-length) fusion protein carrying a mutated S1P cleavage site with or without S2P co-expression in HEK293 cells. Scale bar, 25 μm. **B** Immunoblot analysis for full-length and cleaved (clv) GFP-CREB3L2 or GFP-CREB3L2_S1P_ in HEK293 cells expressing either S1P or S2P. **C** Immunoblot analysis of endogenous CREB3L2 processing in HEK293 cells treated with vehicle (DMSO), thapsigargin (TG), or bortezomib (BTZ) for 5 h; shown are *n* = 2 independent biological replicates. **D** Immunoblot analysis of HRP-tagged, full-length CREB3L2 processing in HEK293 cells treated with Aβ_42_ in the presence of vehicle or nelfinavir (NF) for 2 h. **E** Quantitative immunofluorescence imaging of cortical neurons expressing full-length GFP-CREB3L2 incubated with Aβ_42_ or vehicle (2 h). Cells were pre-treated for 30 minutes with NF or control before Aβ_42_ stimulation. Nuclear-to-cytoplasmic GFP fluorescence ratios are presented as means ± SEM of 138-161 neurons per condition, obtained over *n* = 3 independent biological replicates. Holm-Šídák multiple unpaired t-tests, ****P-value < 0.000001. Scale bar, 25 μm. **F** Immunoblot analysis of nuclear CREB3L2 and ATF4 levels in cortical neurons treated with vehicle (DMSO) or bortezomib (BTZ) in the presence or absence of nelfinavir (NF). Means ± SEM of *n* = 6 independent biological replicates, normalized to HDAC1. Holm- Šídák multiple unpaired t-tests, ****p < 0.0001; *p < 0.05. **G** PLA of CREB3L2-ATF4 in cortical neurons treated with vehicle (DMSO) or bortezomib (BTZ) in the presence or absence of nelfinavir (NF). Means of means ± SEM of *n* = 6 independent biological replicates, 58-60 optical fields per condition. Number of puncta normalized to area of neuronal soma. Holm-Šídák multiple unpaired t-tests, *p < 0.05. **H** Immunoblot analysis of endogenous S2P levels in cortical neurons treated with vehicle (DMSO) or bortezomib (BTZ) obtained over *n* = 5 biological replicates, normalized to β-actin. Same membranes as analyzed in Fig. 1 D and F.

The formation and function of CREB3L2-ATF4 heterodimers requires several distinct steps, including the synthesis (and in the case of CRB3L2 processing) of both transcription factors, their binding, and the translocation of the heterodimer into the nucleus. CREB3L2 is produced as a transcriptionally inactive ER-transmembrane protein that can translocate to the nucleus only after being processed by the ER membrane-bound site-1 and/or site-2 proteases, S1P and S2P, respectively [11]. Previously, we reported that the proteolytic cleavage of CREB3L2 and its subsequent release from the ER membrane are dependent on S2P in dorsal root ganglion neurons [37]. To confirm that S2P cleavage alone is sufficient to release CREB3L2 from the ER membrane in other cells as well, we transfected HEK293 cells with GFP-CREB3L2 carrying a mutated S1P site and then co-expressed cells with S2P. GFP signals were originally reticular in nature (membrane-bound, full-length CREB3L2) but distinctively accumulated in the nucleus with S2P co-expression, indicating that cleavage and activation can occur independently of S1P (Fig. 3A). Likewise, immunoblot analysis demonstrated that expression of S2P, but not S1P, was sufficient to promote the cleavage of CREB3L2 while mutating the S1P cleavage site did not impede its processing, indicating that CREB3L2 activation can occur independently of S1P (Fig. 3B). To investigate the necessity of S2P for the cleavage of endogenous CREB3L2, we used a pharmacological inhibitor of S2P, nelfinavir (NF) [37, 38]. In HEK293 cells, levels of cleaved CREB3L2 were increased by thapsigargin-induced ER stress and proteasome inhibition and treatment with NF abolished this effect (Fig. 3C). Likewise, in HEK293 cells expressing HRP-tagged full-length CREB3L2, the Aβ_42_-induced cleavage of CREB3L2 was reduced in the presence of NF (Fig. 3D). Additionally, treatment with NF prevented the Aβ_42_-induced nuclear localization of CREB3L2 in cortical neurons (Fig. 3E). Together, these data establish that S2P activity is necessary for the nuclear accumulation of CREB3L2 in response to Aβ_42_ exposure.

### Inhibition of the proteasome induces cleavage of CREB3L2 by S2P

To investigate the effects of proteasome inhibition on the subcellular localization of cleaved CREB3L2, we treated neurons with BTZ for 5 h in the presence of NF. The proteasome inhibitor-dependent increase in nuclear CREB3L2 levels was greatly reduced by S2P inhibition (Fig. 3F). NF did not prevent the BTZ-dependent accumulation of CREB3L2-ATF4 heterodimers in neurons, reflecting that S2P-dependent cleavage is not required for dimerization of the transcription factors, just for the localization to the nucleus (Fig. 3G). Next, we analyzed levels of S2P to determine if proteasome inhibition in neurons could contribute to the increased cleaved levels of CREB3L2, and indeed, S2P levels were increased by proteasome inhibition (Fig. 3H). Together, these data establish that proteasome inhibition increases S2P-dependent cleavage of CREB3L2, potentially by increasing the expression S2P.

### CREB3L2 levels are negatively regulated by HRI activation

**Fig. 4:**
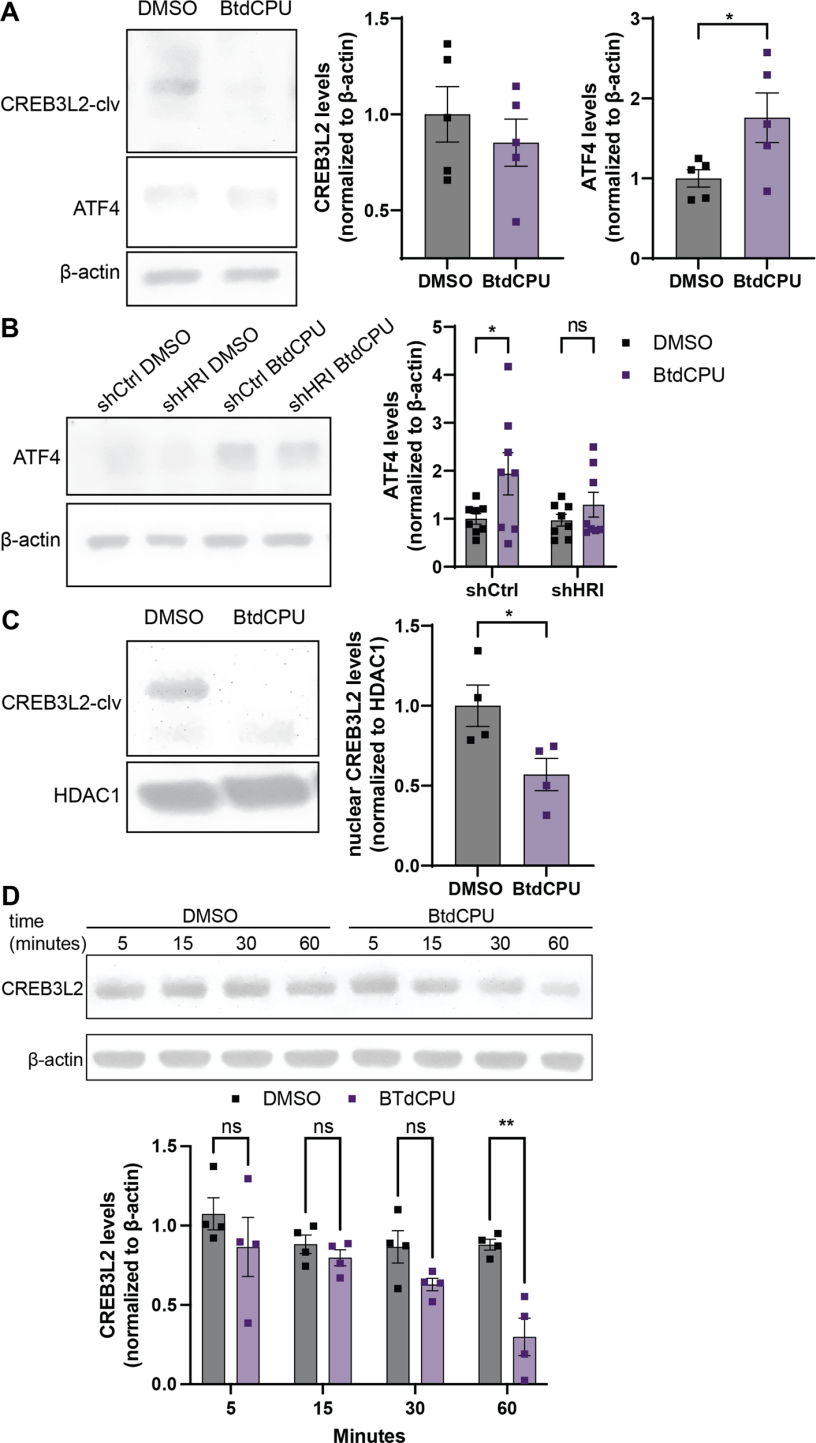
Long-term HRI activation negatively regulates CREB3L2 levels. **A** Immunoblot analysis of CREB3L2 and ATF4 protein levels in whole cell lysates of vehicle (DMSO)- or BtdCPU-treated cortical neurons. Means ± SEM of *n* = 5 independent biological replicates, normalized to β-actin. Unpaired, two-tailed t-test; *p < 0.05. Same membranes as analyzed in Fig. 1 D and F. **B** Immunoblot analysis of ATF4 levels in vehicle (DMSO) or BtdCPU-treated cortical neurons transduced with either shCtrl or shHRI. Means ± SEM of *n* = 8 independent biological replicates. Holm-Šídák multiple unpaired t-tests; *p < 0.05. **C** Immunoblot analysis of nuclear CREB3L2 levels in vehicle (DMSO)- or BtdCPU-treated cortical neurons. Means ± SEM of *n* = 4 biological replicates, normalized to HDAC1. Unpaired, two-tailed t-test; *p < 0.05. **D** Time course analysis of CREB3L2 expression in cortical neurons treated with DMSO or BtdCPU. Means ± SEM of *n* = 4 independent biological replicates. Holm-Šídák multiple unpaired t-tests; **p < 0.01.

So far, our data indicated that proteasome inhibition is crucial for CREB3L2-ATF4 signaling, but the eIF2α kinase controlling translation of *Atf4* and *Creb3l2* remained unidentified. Proteasome inhibition activates HRI-eIF2α signaling [14, 16], and HRI is activated in the presence of amyloid-like protein aggregates and by α-synuclein in non-neuronal cells [39, 40]. To determine the role of HRI activity in regulating levels of ATF4 and CREB3L2 in neurons, we treated cortical neurons with a small molecule activator of HRI, BtdCPU [41]. Treatment with BtdCPU resulted in significantly increased ATF4, but a statistically non-significant decrease is CREB3L2 levels in whole cell lysates (Fig. 4A). To ensure that any effect seen with BtdCPU was mediated by HRI, we transduced cortical neurons with virus expressing non-targeting control or HRI-targeting shRNAs (shCtrl and shHRI, respectively). The increase in ATF4 levels observed with BtdCPU treatment was abolished in shHRI neurons (Fig. 4B). To gain further insight into the role of HRI in regulating the transcriptionally relevant cleaved form of CREB3L2, we analyzed its levels in the nucleus and found that BtdCPU treatment for 5 h resulted not in an increased but instead a significant reduction of CREB3L2 levels in the nucleus (Fig. 4C). To investigate this finding further, we performed a time course experiment and observed that BtdCPU had an effect on CREB3L2 levels in whole cell lysates of cortical neurons only after 60 minutes of treatment (Fig. 4D). Together, our findings identify that HRI activation positively regulates levels of ATF4, but sustained activation reduces levels of CREB3L2.

### CREB3L2 levels are primarily regulated by proteasome-dependent degradation

**Fig. 5:**
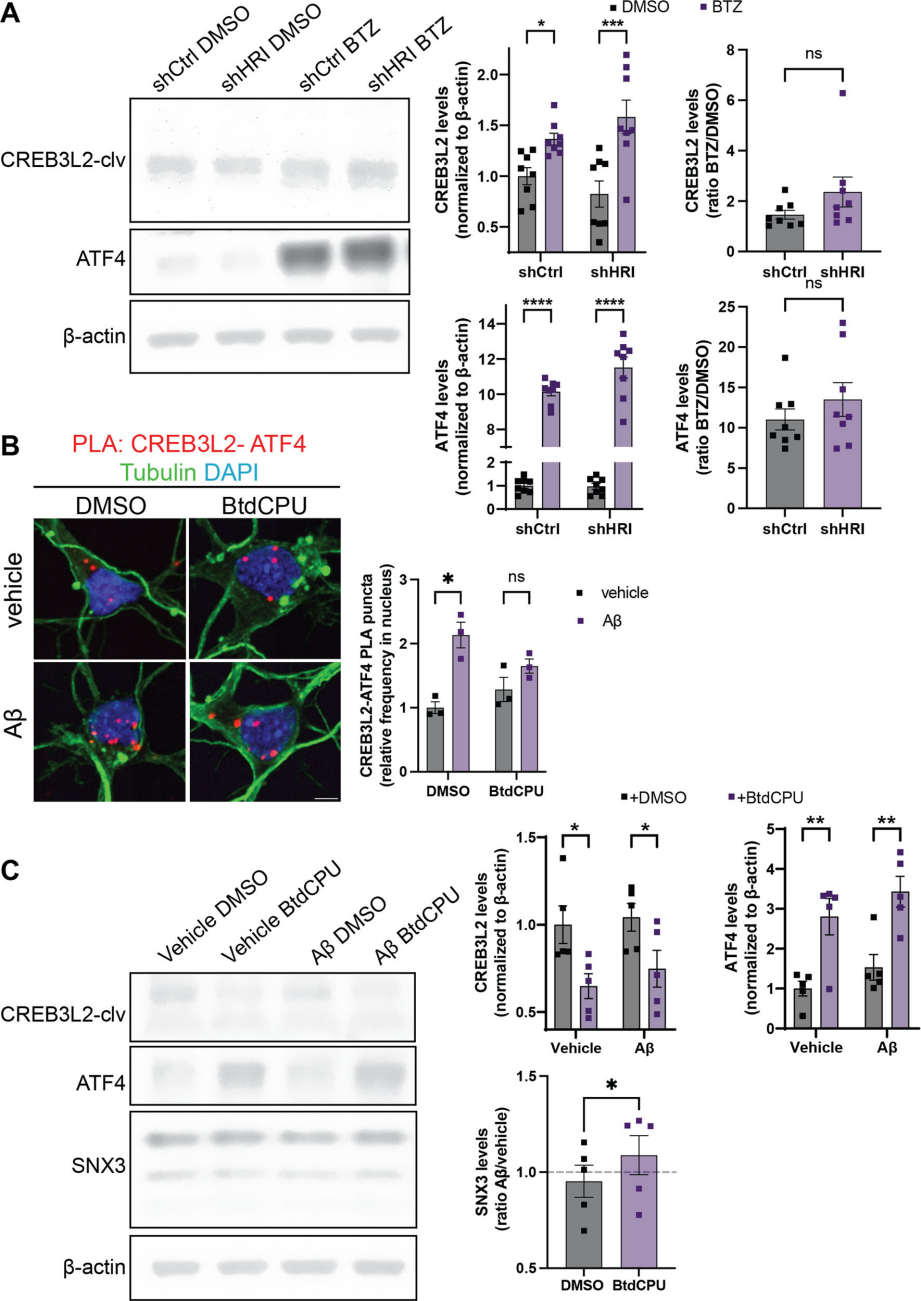
HRI activation interferes with CREB3L2-ATF4 signaling. **A** Immunoblot analysis of CREBL2 and ATF4 in vehicle (DMSO) or bortezomib (BTZ)-treated cortical neurons transduced with either shCtrl or shHRI. Means ± SEM of *n* = 8 independent biological replicates. Two-way ANOVA; *p < 0.05, ***p < 0.001. ATF4 and CREB3L2 ratios of BTZ- to DMSO- treated samples in shCtrl compared to shHRI for each trial. **B** PLA of CREB3L2-ATF4 in cortical neurons treated with DMSO or BtdCPU and Aβ_42_ or vehicle for 8 h. Means of means ± SEM of *n* = 3 independent biological replicates, 62–70 optical fields per condition for nuclear ATF4-CREB3L2 events. Holm- Šídák multiple unpaired t-tests, *p < 0.05. Scale bar, 5 µm. **C** Immunoblot analysis of CREB3L2, ATF4, and SNX3 in whole cell lysates of cortical neurons treated with vehicle or Aβ_42_ in the presence of DMSO or BtdCPU. Means ± SEM of *n* = 5 independent biological replicates. Holm-Šídák multiple unpaired t-tests; *p < 0.05, **p < 0.01. SNX3 ratios of Aβ_42_- over vehicle-exposed neurons compared between DMSO- and BtdCPU-treated. Paired t-test, two-tailed. *p < 0.05.

A possible explanation for the seemingly contradictory finding that HRI activation results in increased ATF4 expression but decreases CREB3L2 levels, might be that CREB3L2 gets rapidly degraded via the ubiquitin-proteasome pathway [21]. To distinguish between the effects of proteasome inhibition and HRI activation, we measured the effect of proteasome inhibition on CREB3L2 and ATF4 levels in neurons expressing shCtrl or shHRI. Levels for both transcription factors were significantly increased in the BTZ conditions (Fig. 5A). The knockdown of HRI did not significantly change the increase for either transcription factor. Collectively, these findings reveal that levels of CREB3L2 are controlled by proteasome-dependent degradation, while sustained HRI activity is negatively correlated with CREB3L2 levels.

### HRI activation prevents CREB3L2-ATF4 signaling effects

Because HRI negatively regulates CREB3L2 levels, we reasoned that activation of HRI during Aβ_42_-induced stress might rescue some of the CREB3L2-ATF4 effects. To directly test this possibility, we applied BtdCPU to Aβ_42_-treated neurons. Using PLA, we found that BtdCPU abolished the Aβ_42_-dependent increase in the number of CREB3L2-ATF4 heterodimers in the nuclei of cortical neurons (Fig. 5B). CREB3L2 levels were significantly reduced in control and Aβ_42_-treated neurons co-treated with BtdCPU, while ATF4 levels significantly increased in either condition (Fig. 5C). A transcriptional effect of CREB3L2-ATF4 signaling is the reduction of SNX3 expression, a component of the retromer complex [6]. Activation of HRI in Aβ_42_-treated neurons prevented this effect and instead reverted SNX3 expression to baseline levels (Fig. 5C). These findings suggest that HRI activation by BtdCPU prevents the Aβ_42_-induced accumulation of the ATF4-CREB3L2 heterodimer and its associated transcriptional effects.

## Discussion

Here, we report that the formation of the AD-linked transcription factor heterodimer CREB3L2-ATF4 is downstream of the inhibition of the proteasome by oligomeric Aβ_42_. The finding that eIF2α phosphorylation in the absence of proteasome inhibition is insufficient to induce CREB3L2-ATF4, led us to investigate the role of the eIF2α-kinase HRI, which can be activated by proteasome inhibition. ATF4 levels were increased by BTZ-treatment even in the context of HRI knockdown. While this finding is formally in line with the notion that BTZ could activate other eIF2ɑ kinases, prior work has identified HRI as the primary kinase mediating proteasome inhibition-dependent eIF2α [14, 16]. We found that CREB3L2 is negatively regulated by sustained, i.e., longer than 60 minutes activation of HRI. Importantly, we did not observe a reduction in CREB3L2 with shorter times of HRI activation, leaving the possibility open that short-term activation of HRI or HRI activation in the context of proteasome inhibition can induce CREB3L2 levels. The fact that levels of both CREB3L2 and the heterodimer were reduced with HRI activation suggests that the amount of available CREB3L2 might be rate-limiting for heterodimer formation. These findings are compatible with a model in which both ATF4 and CREB3L2 are translated downstream of HRI, but in the absence of proteasome inhibition, CREB3L2 is rapidly degraded, and either no heterodimer is formed or it is unstable. In this model, the reduction of CREB3L2 levels under prolonged HRI activity would be part of a self-limiting stress response. This safeguarding mechanism fails in neurons exposed to Aβ_42_, in a situation where eIF2α phosphorylation and proteasome inhibition coincide.

The mechanisms by which direct, prolonged activation of HRI lowers levels of CREB3L2 while proteasome inhibition results in increased levels of CREB3L2 remain to be explored. Our finding that proteasome inhibition increases S2P levels and S2P-dependent release of CREB3L2 from the ER neurons suggests that this step might be involved in preventing the rapid degradation of CREB3L2.

In conclusion, we set out to elucidate the signaling pathways responsible inducing the AD-specific transcription factor complex, CREB3L2-ATF4, in response to soluble oligomeric Aβ_42_. Our results establish that Aβ_42_ inhibits the proteasome, resulting in increased levels of both transcription factors in the nucleus. We provide evidence that the eIf2α-kinase HRI regulates levels of ATF4 and CREB3L2 differently, where prolonged HRI activation results in increased levels of ATF4 and decreased levels of CREB3L2. However, with proteasome inhibition, which can activate the HRI-eIf2α axis, levels CREB3L2 are increased as well. This data, along with previous literature suggests that CREB3L2 levels are usually kept low under normal physiological conditions due to basal level of HRI activity [16], then under proteasome inhibition, CREB3L2 levels increase because of lack of degradation and possibly through increased levels of the protease responsible for producing active CREB3L2. In response to soluble oligomeric Aβ_42_, the effects of proteasome inhibition on CREB3L2 and heterodimer levels outweigh those of simple HRI activity. However, the overactivation of HRI in neurons treated with Aβ_42_ can counteract some of the heterodimer-dependent transcriptional changes. These data suggest activation of HRI during the accumulation of misfolded proteins/proteasome inhibition as a potential approach to correct gene expression changes in response to Aβ_42_.

## Supplementary information

Original Western Blots

## Data Availability

The data supporting the findings of this study are available from the corresponding author upon reasonable request.
