# Supplementary figures and images for: Modulation of CREB3L2-ATF4 heterodimerization via proteasome inhibition and HRI activation in Alzheimer’s disease pathology
Source: Cell Death Dis. 2025 Mar 31;16(1):225. doi: 10.1038/s41419-025-07586-0 (PMC11958753; doi:10.1038/s41419-025-07586-0)

**Fig. 1C**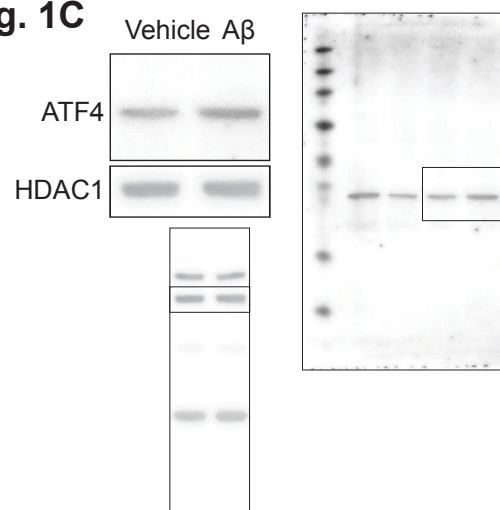**Fig. 1D**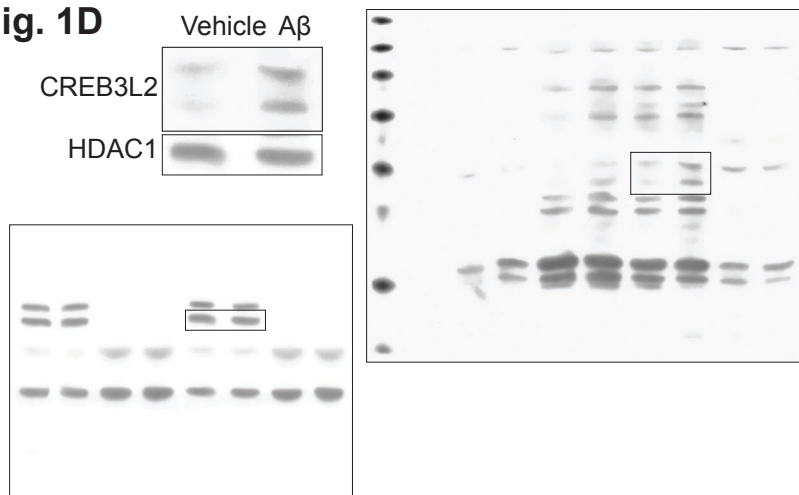**Fig. 1F**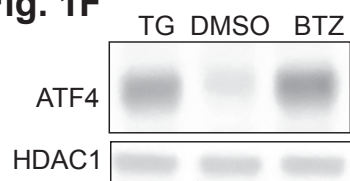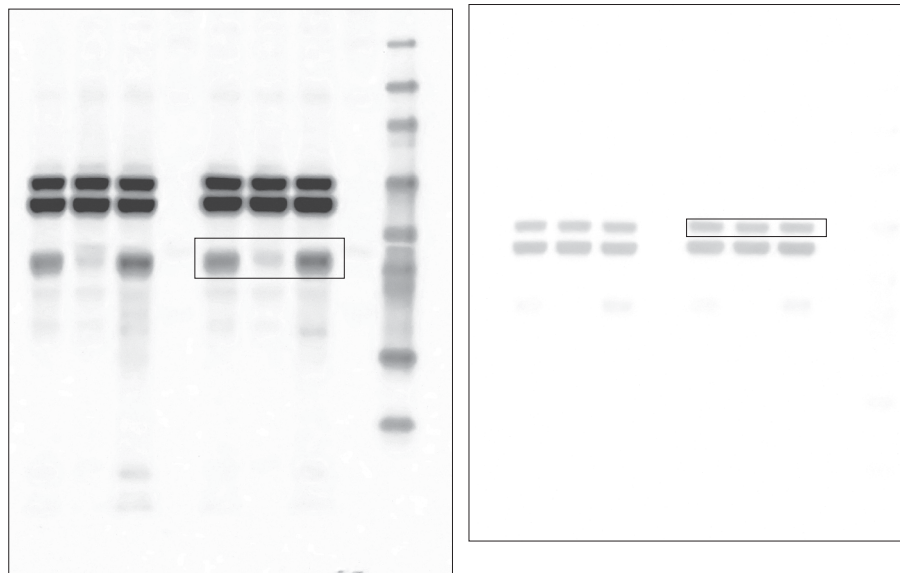**Fig. 1G**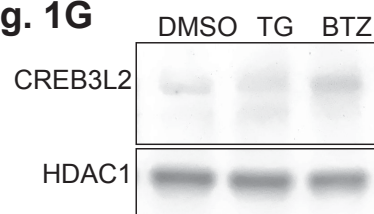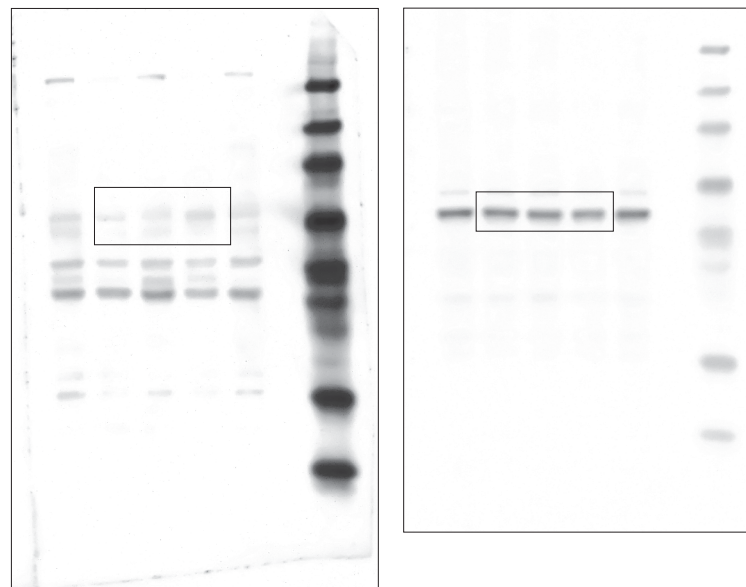**Fig. 1I**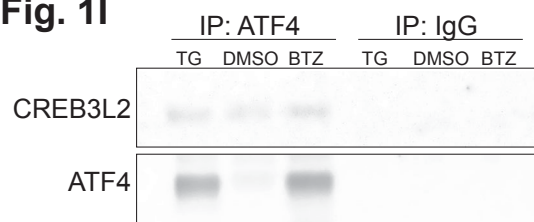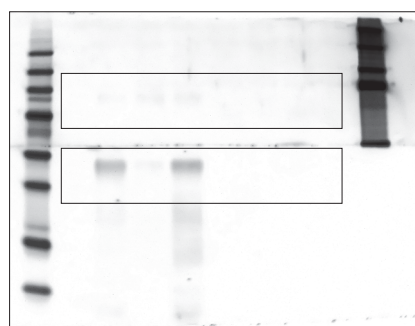

Fig. 3B

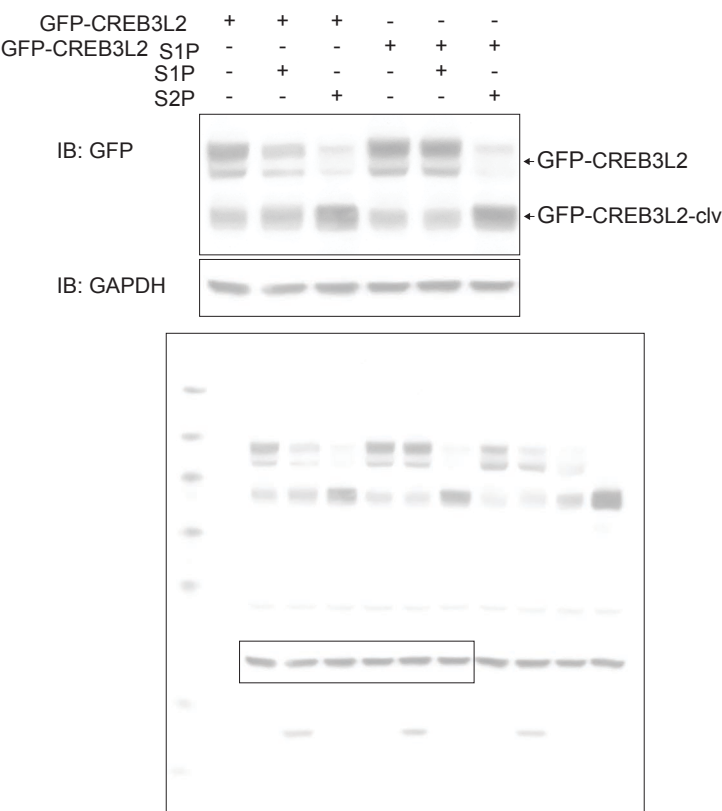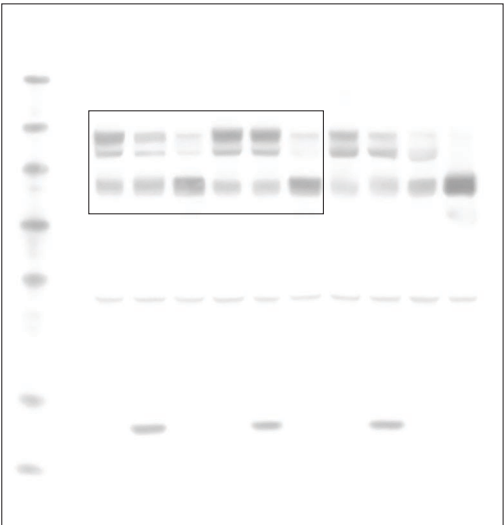

Fig. 3C

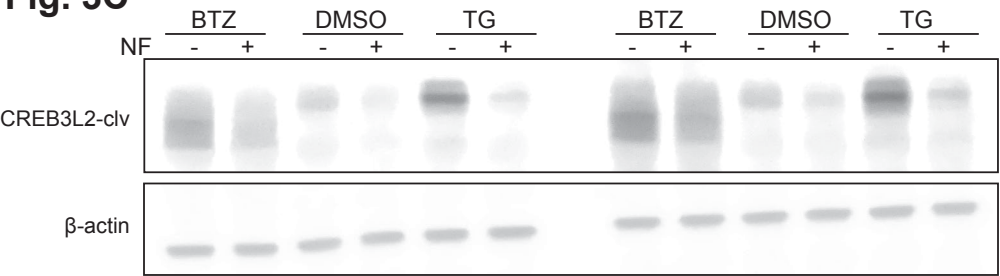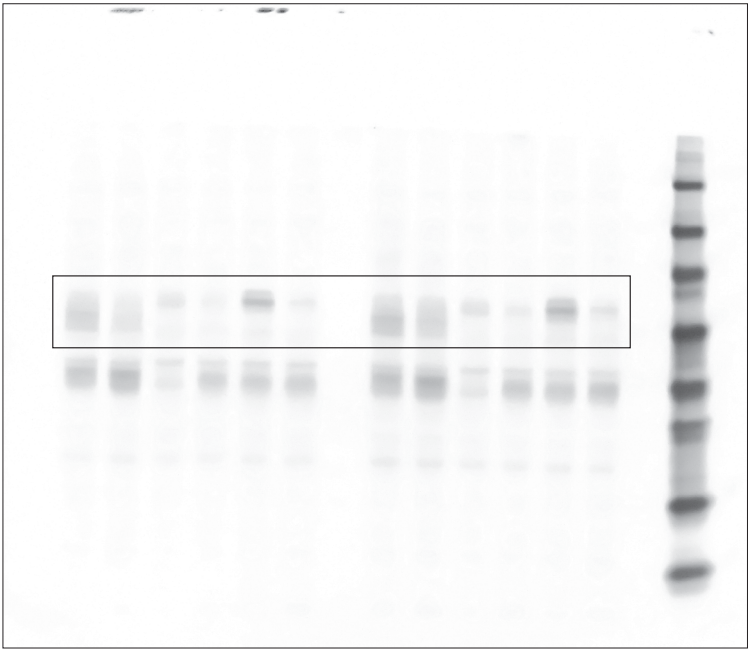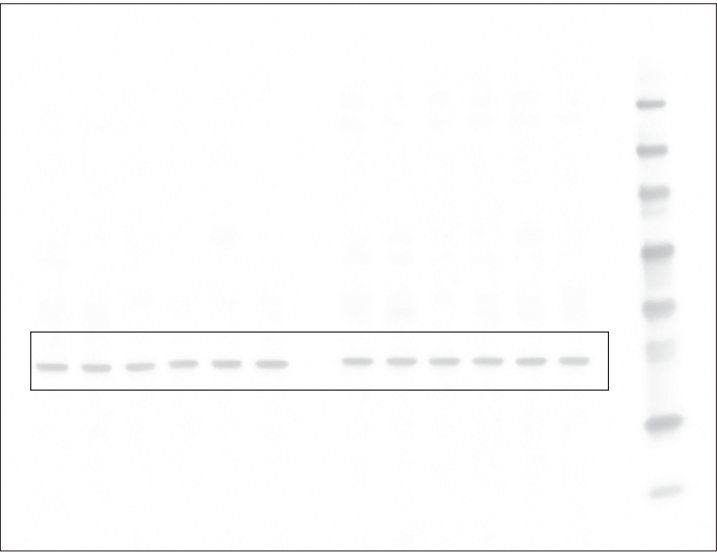

**Fig. 3D**

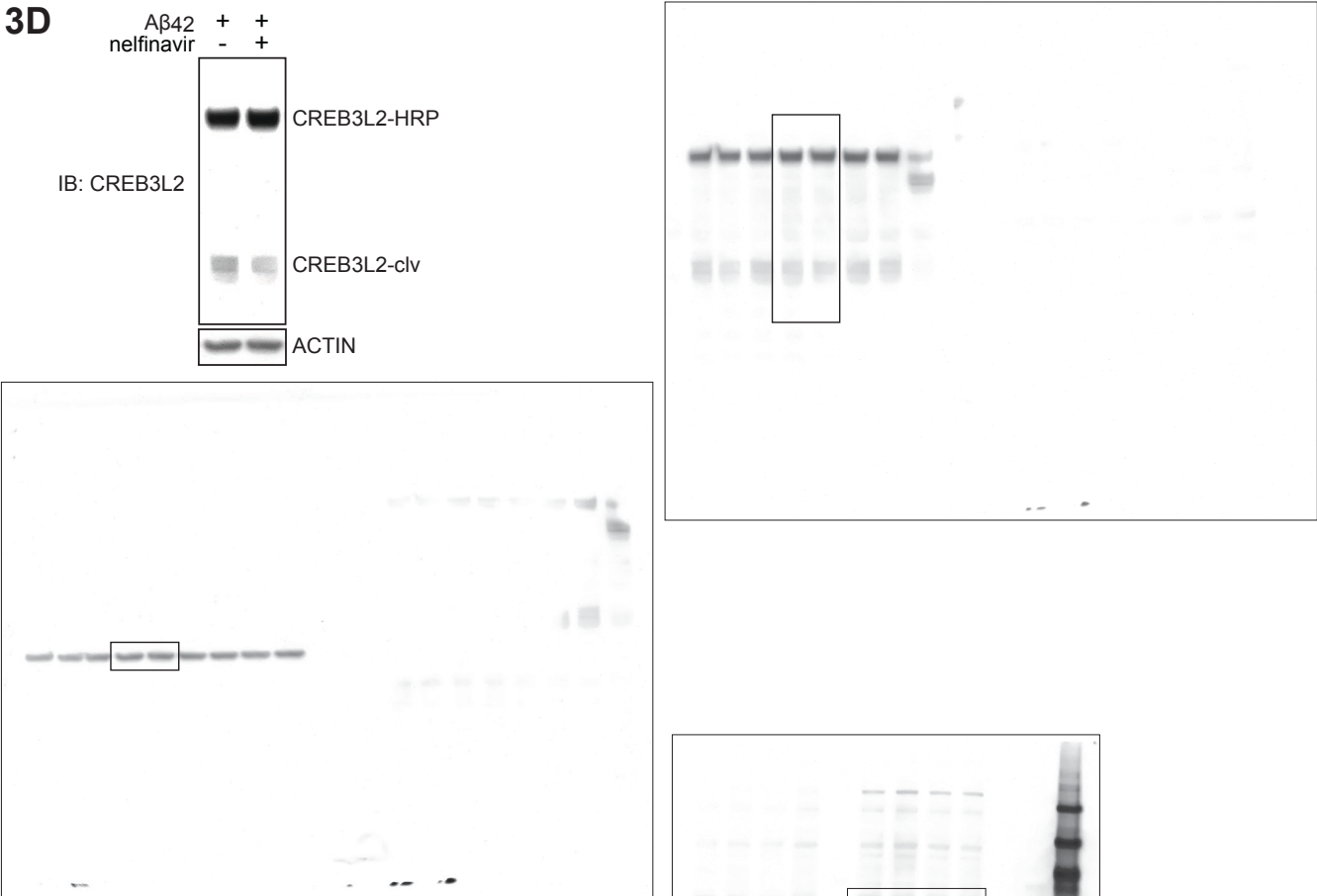

**Fig. 3F**

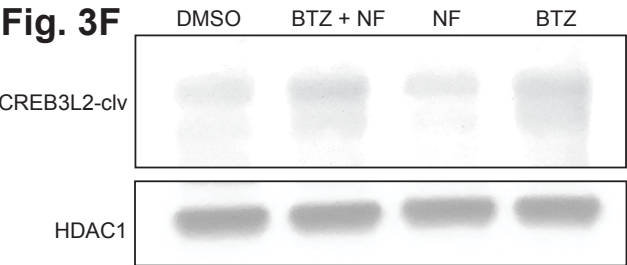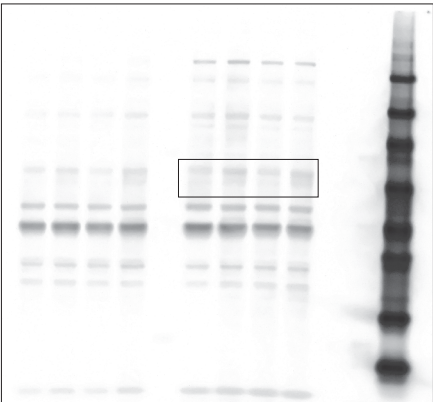

**Fig. 3H**

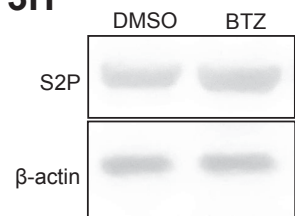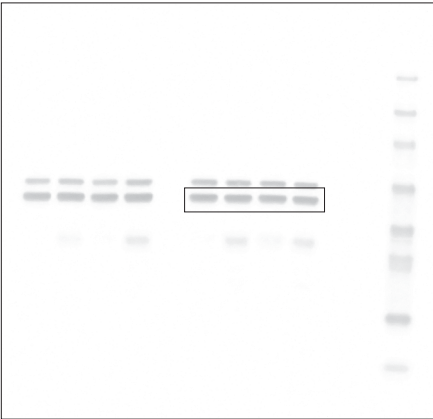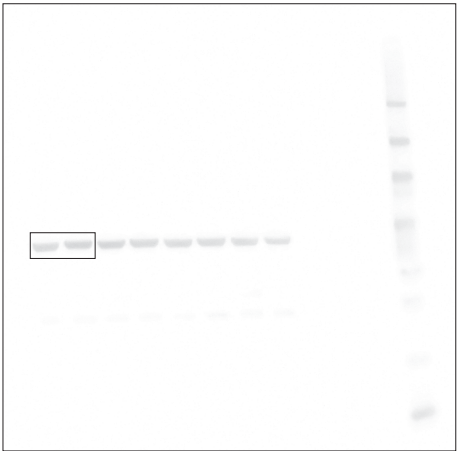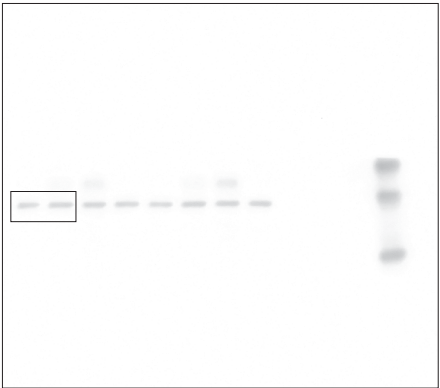

**Fig. 4A**

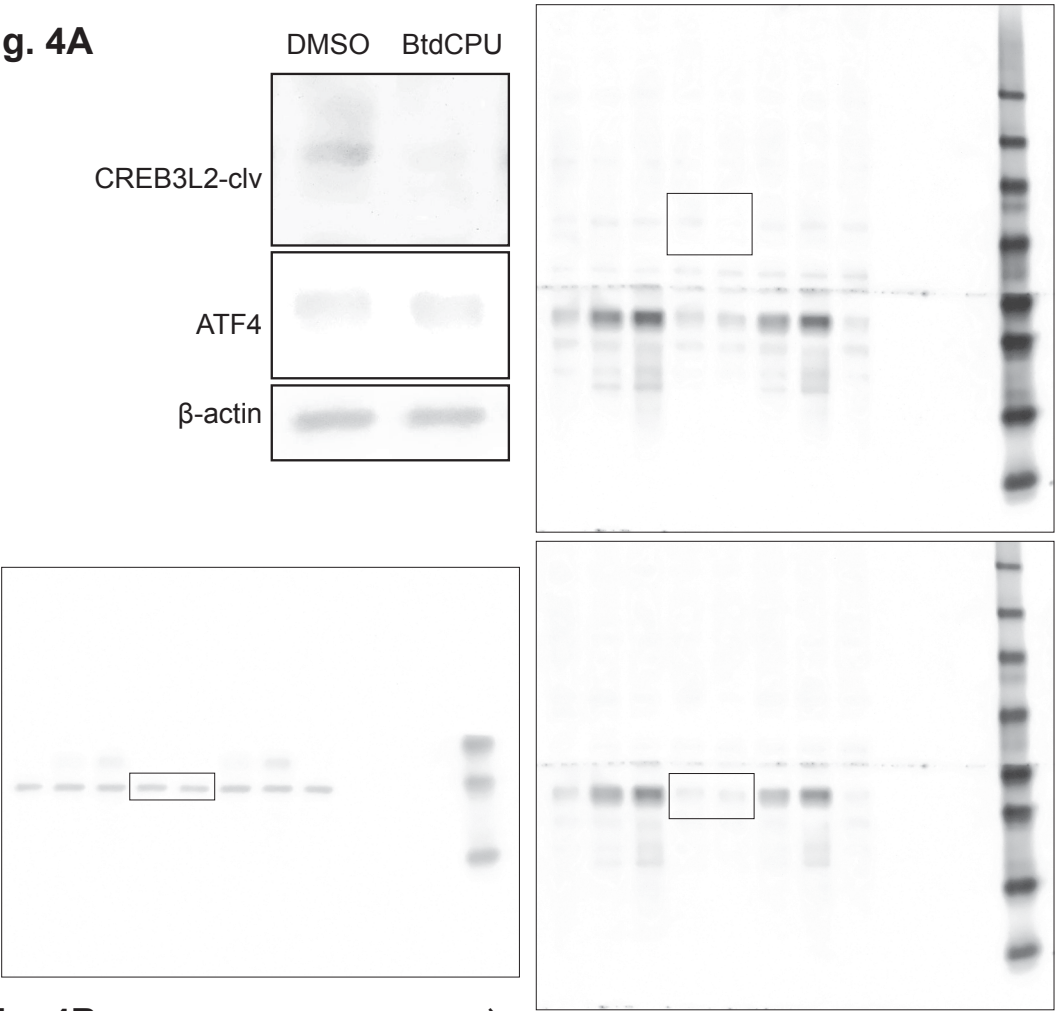

**Fig. 4B**

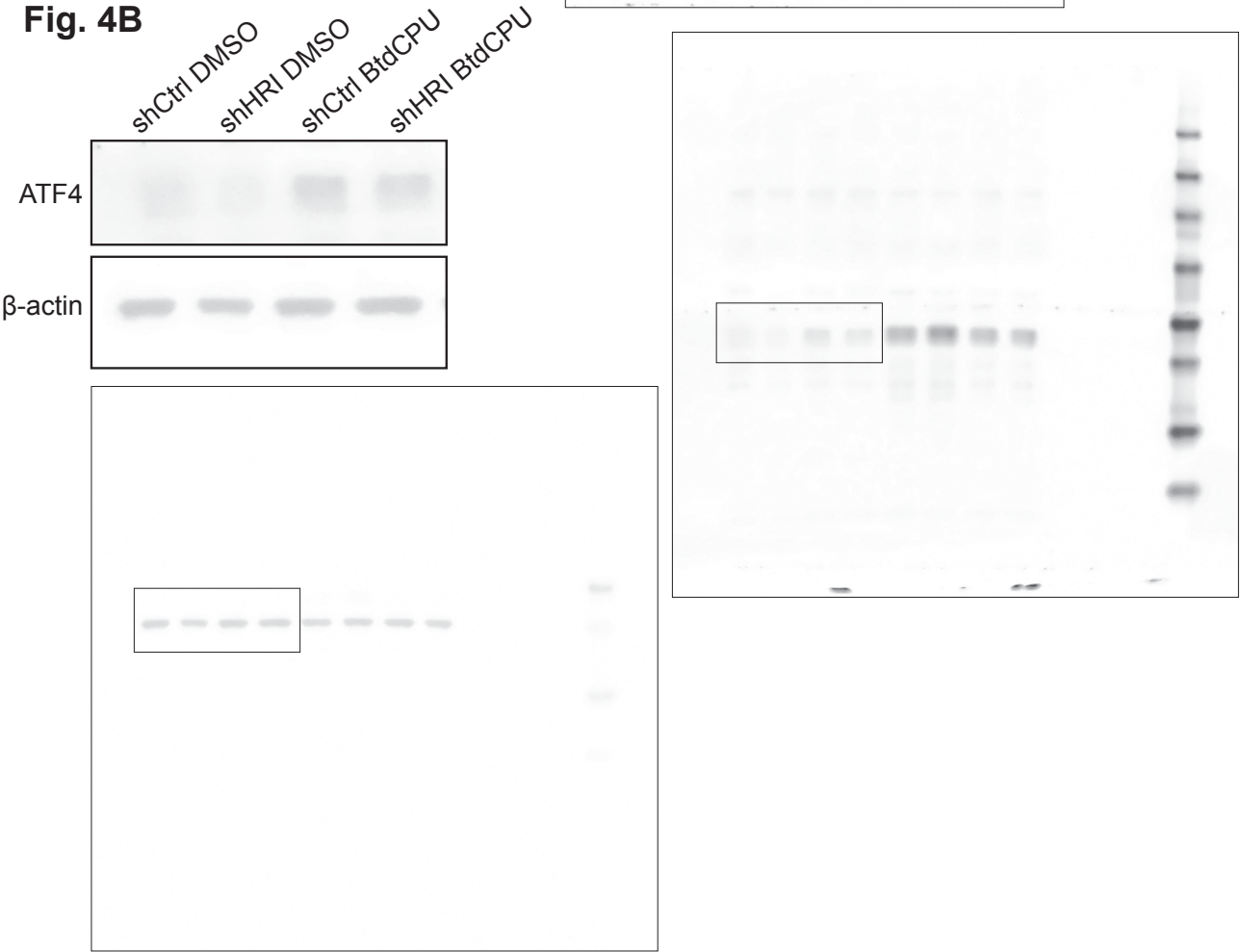

**Fig. 4C**

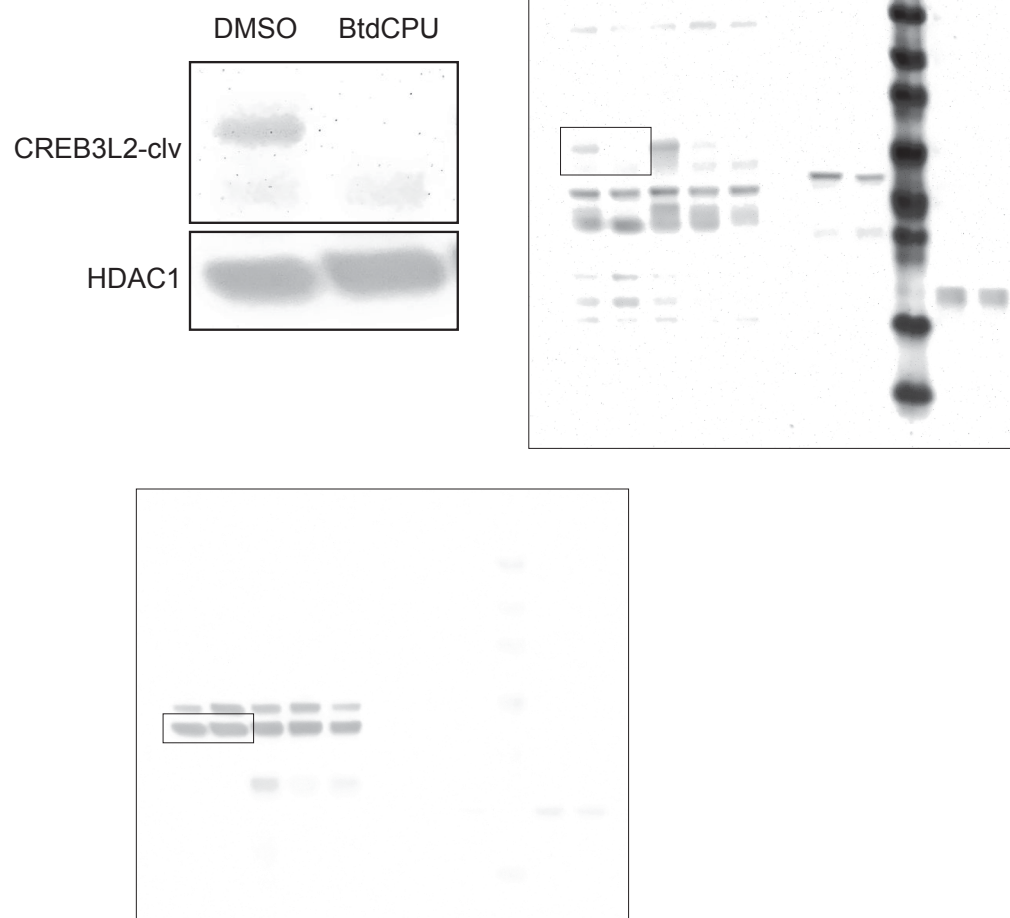

**Fig. 4D**

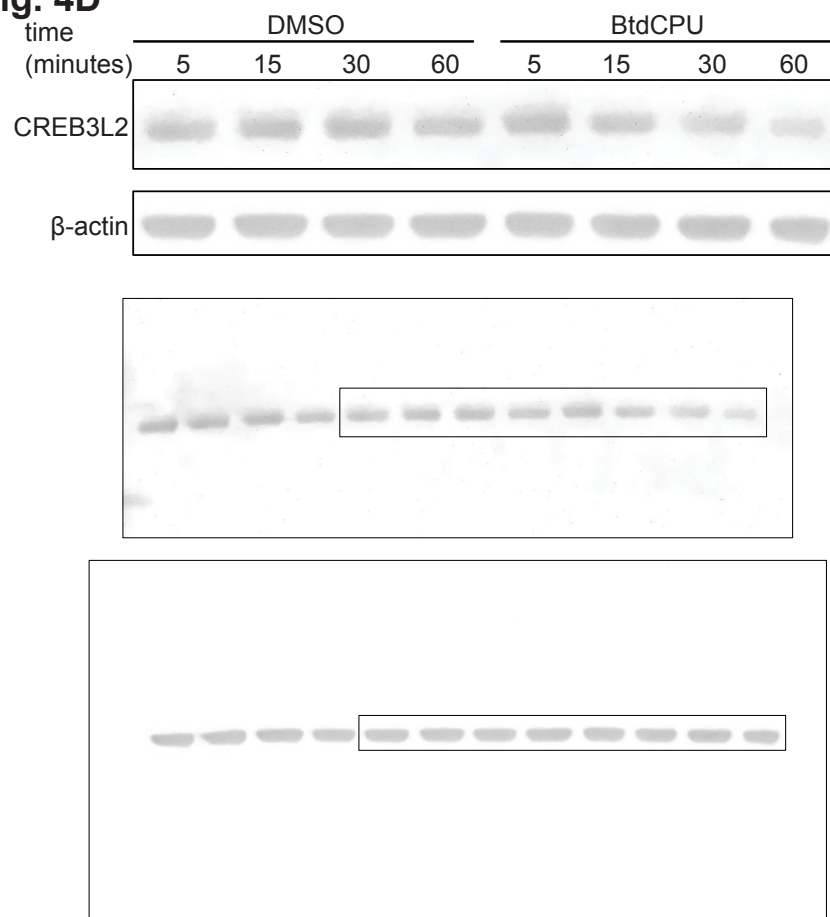

**Fig. 5A**

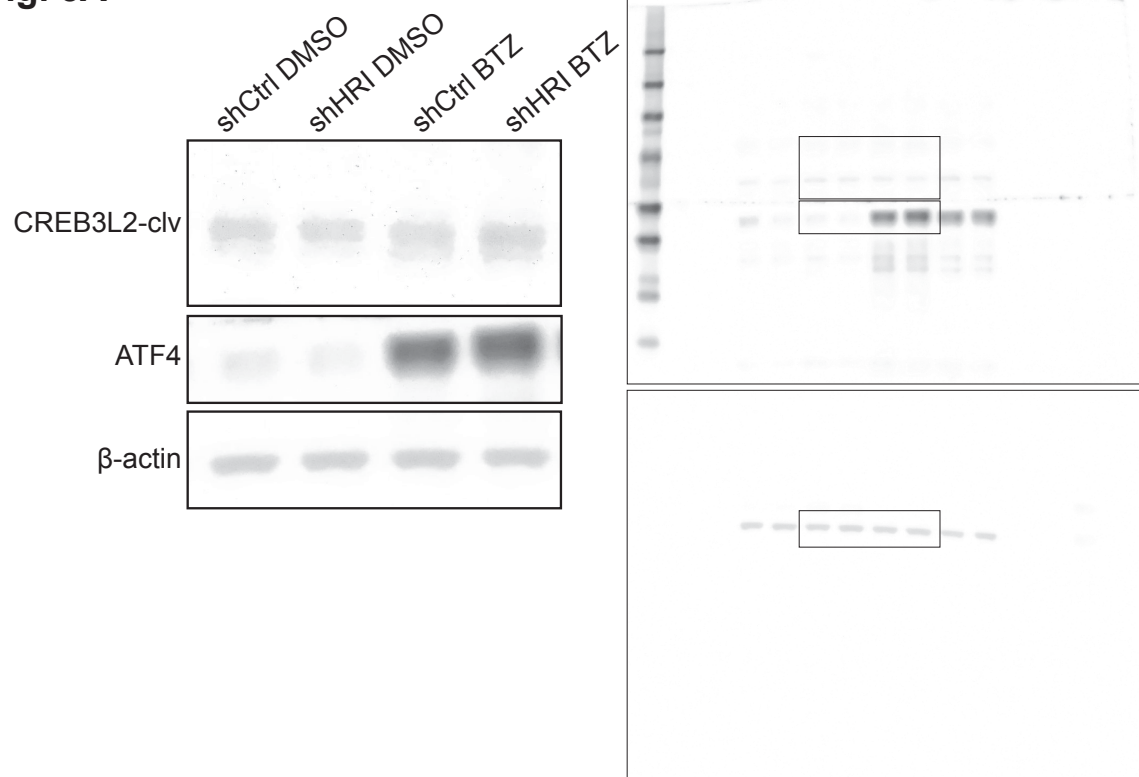

**Fig. 5C**

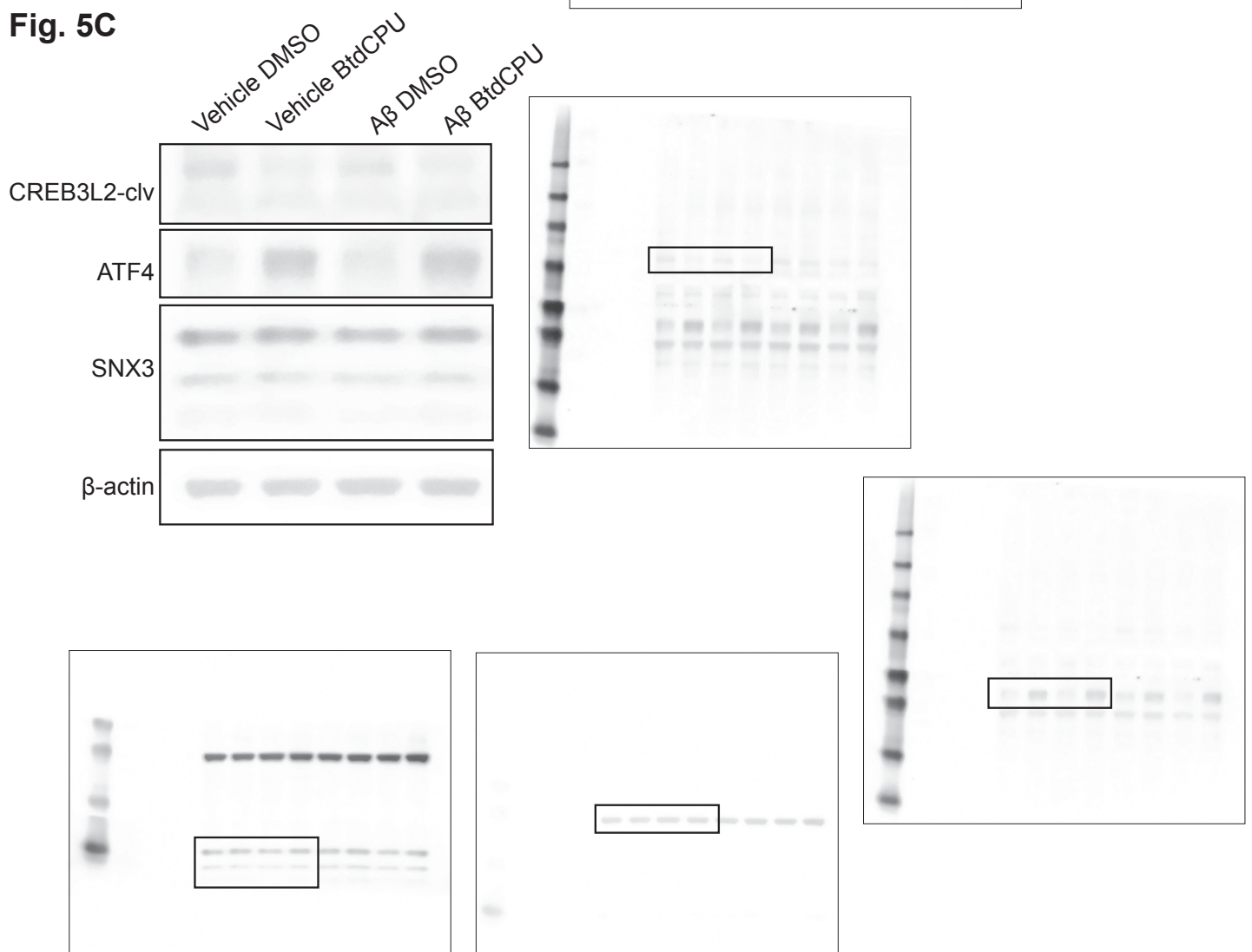

Supplement: Supplementary file 1 — Original Western Blots [file 41419_2025_7586_MOESM1_ESM.pdf]
